# Forward Modeling Reveals Multidecadal Trends in Cambial Kinetics and Phenology at Treeline

**DOI:** 10.3389/fpls.2021.613643

**Published:** 2021-01-28

**Authors:** Jan Tumajer, Jakub Kašpar, Hana Kuželová, Vladimir V. Shishov, Ivan I. Tychkov, Margarita I. Popkova, Eugene A. Vaganov, Václav Treml

**Affiliations:** ^1^Department of Physical Geography and Geoecology, Faculty of Science, Charles University, Prague, Czechia; ^2^Institute of Botany and Landscape Ecology, University of Greifswald, Greifswald, Germany; ^3^Laboratory for Integral Studies of Forest Dynamics of Eurasia, Siberian Federal University, Krasnoyarsk, Russia; ^4^Sukachev Institute of Forest SB RAS, Krasnoyarsk, Russia; ^5^Rectorate, Siberian Federal University, Krasnoyarsk, Russia; ^6^Center for Forest Ecology and Productivity of the Russian Academy of Sciences, Moscow, Russia

**Keywords:** cambial phenology, dendrochronology, growing season, process-based modeling, treeline, VS-model, xylogenesis

## Abstract

Significant alterations of cambial activity might be expected due to climate warming, leading to growing season extension and higher growth rates especially in cold-limited forests. However, assessment of climate-change-driven trends in intra-annual wood formation suffers from the lack of direct observations with a timespan exceeding a few years. We used the Vaganov-Shashkin process-based model to: (i) simulate daily resolved numbers of cambial and differentiating cells; and (ii) develop chronologies of the onset and termination of specific phases of cambial phenology during 1961–2017. We also determined the dominant climatic factor limiting cambial activity for each day. To asses intra-annual model validity, we used 8 years of direct xylogenesis monitoring from the treeline region of the Krkonoše Mts. (Czechia). The model exhibits high validity in case of spring phenological phases and a seasonal dynamics of tracheid production, but its precision declines for estimates of autumn phenological phases and growing season duration. The simulations reveal an increasing trend in the number of tracheids produced by cambium each year by 0.42 cells/year. Spring phenological phases (onset of cambial cell growth and tracheid enlargement) show significant shifts toward earlier occurrence in the year (for 0.28–0.34 days/year). In addition, there is a significant increase in simulated growth rates during entire growing season associated with the intra-annual redistribution of the dominant climatic controls over cambial activity. Results suggest that higher growth rates at treeline are driven by (i) temperature-stimulated intensification of spring cambial kinetics, and (ii) decoupling of summer growth rates from the limiting effect of low summer temperature due to higher frequency of climatically optimal days. Our results highlight that the cambial kinetics stimulation by increasing spring and summer temperatures and shifting spring phenology determine the recent growth trends of treeline ecosystems. Redistribution of individual climatic factors controlling cambial activity during the growing season questions the temporal stability of climatic signal of cold forest chronologies under ongoing climate change.

## Introduction

The seasonal cycle of global temperature shows prominent year-to-year variability and low-frequency trends in phase (timing of seasons) and amplitudes (Mann and Park, 1996; Stine et al., 2009; Cornes et al., 2017). This variance is reflected in intra-annual dynamics of climatically driven biological processes. For example, significant variability and/or trends have been observed in leaf phenology (Cleland et al., 2007; Chen et al., 2019; Jin et al., 2019), transpiration (Oishi et al., 2010), photosynthesis (Xu et al., 2016), and net ecosystem productivity (Froelich et al., 2015). The dynamics of cambial activity form one of the most important seasonally related processes (Vaganov et al., 2006), as their intra-annual pattern is tightly related to the carbon sequestration cycle and to carbon fluxes of entire forest ecosystem (Cuny et al., 2015; Friend et al., 2019). Compared to biological processes mentioned above, data on seasonal cambial dynamics usually span only short periods, typically 1–10 years (Globoxylo Initiative^1^), posing significant uncertainty in quantification of forest responses to climatic variability.

Northern and high-elevation forests experience prominent year-to-year changes in cambial dynamics (process of xylem tissue development), phenology (timing of cambial activity) and kinetics (speed of xylem cell differentiation). The initiation of cambial activity in cold regions is driven mainly by temperature (Rossi et al., 2008; Delpierre et al., 2019; Huang et al., 2020). Consequently, the date of cambial activity onset tightly follows the variability of spring temperature around specific thresholds (Rossi et al., 2007; Treml et al., 2015). Similarly, onset (Rossi et al., 2016), rate and duration (Deslauriers and Morin, 2005; Deslauriers et al., 2008) of subsequent cell development phases vary from year to year. The progress of tree-ring width and density formation is mostly controlled by dynamics of cell enlargement (Cuny et al., 2014), which at treeline is driven by temperature patterns during late spring-early summer (Castagneri et al., 2017). Climate change-driven shifts in intra-annual growth dynamics modify temporal windows of tree growth sensitivity to climate (Carrer et al., 2017), which has been suggested to be one of the possible drivers of weakening coherence between temperature and growth trends in cold regions (D’Arrigo et al., 2008; Ponocná et al., 2018). Unfortunately, short observations of cambial dynamics in cold regions limit our understanding of trends and inter-annual changes of cambial dynamics at multi-decadal timescales.

This lack of long-term data on trends in cambial dynamics is especially unwelcome in areas where forests have shown significant trends in tree growth. For instance, most stem increment chronologies from mountain treelines in Central Europe show significant increases in growth coupled with increasing temperatures (Büntgen et al., 2006; Ponocná et al., 2016; Björklund et al., 2019; Wieser et al., 2019). Without sufficiently long series of intra-annual growth dynamics it is not possible to determine whether this increase is driven by longer growing seasons, higher growth rates or both. Although greater importance of growth rate for annual growth has been reported by some studies (Prislan et al., 2019; Ren et al., 2019), these results were obtained for limited numbers of years, and it is questionable whether the same characterization would apply to multi-decadal periods.

The lack of long direct cambial dynamics observations can be overcome by application of climate-driven, process-based models of wood formation. They represent mathematical approaches that decompose annually resolved dendrochronological proxies (e.g., tree-ring widths) into intra-annually resolved growth components (Guiot et al., 2014). The current generation of complex process-based models is able to simulate cambial dynamics with a very high precision from the set of experimentally measured environmental and physiological variables (Cabon et al., 2020; Peters et al., 2020). However, input data requirements limit their applicability across large spatial gradients and for long temporal scales. By contrast, models of intermediate complexity provide acceptable precision and their use is not restricted by physiological data availability (Misson, 2004; Vaganov et al., 2006). At the annual scale, their previous applications have included quantification of climatic factors limiting tree-growth (Breitenmoser et al., 2014; Sánchez-Salguero et al., 2017; Tumajer et al., 2017; Rezsöhazy et al., 2020), identification of climatic conditions leading to formation of pointer years (He et al., 2017; Tychkov et al., 2019) and isolation of climatically driven parts of chronology variability (Rydval et al., 2016). At the intra-annual scale, process-based models have mostly been used to reveal patterns of cambial growth and productivity during the year (Touchan et al., 2012; Gennaretti et al., 2017), simulate tracheid size distribution (Popkova et al., 2018) or reconstruct and predict time series of key dates of tree-ring formation (Yang et al., 2017; He et al., 2018a). Although process-based models provide outputs with both annual and intra-annual resolutions, in most studies they were calibrated and validated considering only the annual scale, i.e., observed tree-ring width chronology was compared with simulated tree-ring growth curve (Shishov et al., 2016; Tumajer et al., 2017). By contrast, verification of simulated phenological dates is often based on a single year of observations or is not performed at all due to the lack of available data. Consequently, our understanding of model limitations for specific species and regions is much better for annually resolved outputs than intra-annual outputs.

In this study, we aim to address the lack of validation of intra-annual performance of process-based models and assess the importance of intra-annual growth rates and phenology for annual growth trends. We used the Vaganov-Shashkin model (further abbreviated VS-model), which is a sink-oriented process-based model applicable to conifers, simulating kinetics (growth, division, and differentiation) of cambium and its derivatives aligned in a radial cell file purely from daily resolved climatic data (Vaganov et al., 2006; Anchukaitis et al., 2020). We focused on two research questions: (i) What is the precision of the VS-model simulations of cambial dynamics? and (ii) How did the cambial dynamics at the treeline respond to climate change during last five decades? For addressing the first question, 8 years of xylogenesis monitoring of Norway spruce was used to verify intra-annual dynamics predicted by the VS-model. We assumed that the VS-model has greater ability to represent the first part of the growing season than the second part, because the model does not consider non-climatic drivers that increasingly affect cambial activity toward the end of the growing season, e.g., hormonal concentrations and non-structural carbohydrates dynamics (Hartmann et al., 2017; Furze et al., 2019). To answer the second question, we simulated time series for specific key dates of cambial phenology and growth rates and analyzed their long term-trends. We hypothesized that similarly to climatologically defined growing season length (Dubicka and Głowicki, 2000), the duration of the growth period of treeline Norway spruce has been increasing since the 1960s. We also expected that significant trends toward higher spring-summer growth rates of Norway spruce already documented for lower elevations (Tumajer et al., 2017) will also be found for the treeline.

## Materials and Methods

### Study Area

**FIGURE 1:**
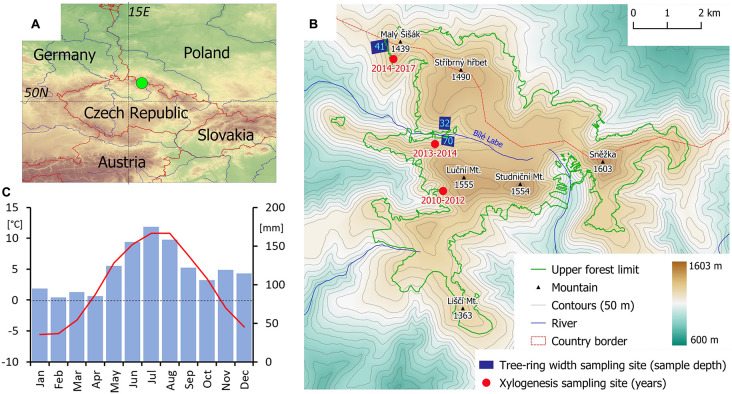
Location of the study area in the Central Europe **(A)**; distribution of individual tree-ring width and xylogenesis sampling sites in local treeline ecotone **(B)**; and monthly temperature means (red) and precipitation sums (blue) **(C)**. Position of upper forest limit (lower boundary of treeline ecotone) according to Treml and Migoń (2015).

The Krkonoše Mts. are situated in the north of the Czechia (N50.75, E15.75; Figures 1A,B). The highest peak (Sněžka Mt.) reaches 1603 m in elevation. The treeline ecotone, formed exclusively of Norway spruce (*Picea abies* (L) Karst.), has an upper margin situated at elevations 1290–1450 m (Treml and Migoń, 2015). The mean annual temperature at the summit areas is about 0.5°C and the total annual precipitation is approximately 1600 mm (Figure 1C). Mean annual temperature increased by 0.28°C/decade during 1961–2017 (Supplementary Figure S1A). The most significant increases in mean monthly temperatures were observed during May, July, and August (0.42–0.46°C/decade). Inter-annual variability (i.e., standard deviation) of mean monthly temperatures is greatest in winter (1.9–2.5°C) and spring (1.7–2.2°C; Supplementary Figure S1B).

### Tree-Ring Data Collection

To establish a regional chronology of tree-ring widths, we cored 143 trees distributed in the treeline ecotone of the Krkonoše Mts. We included trees growing directly at locations where wood formation has been monitored (see below). One core from each tree was extracted using Pressler corer at the height of 1 m from the stem base. All cores were mounted on wooden slices and sanded using progressively finer sandpapers to enhance visibility of tree-ring borders. Tree-ring widths were measured and visually and statistically (synchronicity index, *t*-value) cross-dated using a TimeTable measurement stage (VIAS, Austria) and PAST4 software (Knibbe, 2004). Each series was detrended by fitting a 100-year spline (≈ mean series length) with 50% frequency cut-off (Cook and Peters, 1981). Finally, a regional standard (containing temporal autocorrelation) and residual chronologies (autocorrelation removed in a pre-whitening process) were built by robust averaging of all series. The common signal retained in the chronology was quantified using expressed population signal (EPS)–a measure of the similarity between a given tree-ring chronology and a hypothetical chronology that had been infinitely replicated from the individual radii (Wigley et al., 1984)–and mean inter-series correlation (Rbar) (Cook and Kairiukstis, 1990). All steps of the statistical processing of tree-ring data were performed in R (R Core Team, 2020) using the package ‘dplR’ (Bunn, 2008).

The monitoring of cambial phenology (xylogenesis) took place during the 2010–2017 growing seasons (Treml et al., 2015, 2019). The sampling was performed using Trephor (Rossi et al., 2006) in intervals spanning on average 8 days between April and October/November. The microcores were collected from a 30-cm-wide zone of stem at about 1 m stem height. Different trees (6–9 per year) were sampled each year to avoid the effect of damage caused by previous years’ sampling. Specific sampling microsites (Supplementary Table S1) inside treeline region in a distance of less than 2 km from each other were sampled each year (Figure 1 and Supplementary Figure S2). However, there were non-significant differences in dendrometric parameters of sampled trees (Supplementary Table S2) and cambial phenology and kinetics between sampling microsites (Supplementary Table S3 and Supplementary Figure S3). This indicates low variability of cambial activity dynamics over the study region and, consequently, we treated all xylogenesis data as single dataset representative of the entire treeline.

The laboratory processing of microcores followed standard wood anatomical protocol (Rossi et al., 2006; Gärtner and Schweingruber, 2013) to produce thin (15 μm) stained cross-sections (for details, see e.g., Treml et al., 2015). Each cross-section was observed using transmitted and polarized light under a microscope (Leica, Germany) with magnification up to 400× to count the number of tracheids and assess the developmental stage of each tracheid in terms of its shape, dimensions and progress of cell-wall lignification. We followed the dynamics of cambial activity and tracheid differentiation and distinguished cambial zone cells (CZ), cell enlarging cells (EN), wall thickening cells (WT), and mature (MC) tracheids (Rossi et al., 2003; Rathgeber et al., 2016). Tracheid counting and development assessment were performed for three tracheid radial files in each tree ring.

### Climatic Data

We obtained daily resolved climatic data (mean temperature and total precipitation) for the Szrenica climate station, located approximately 10 km from our sampling sites in a similar mountain environment (N50.79, E15.51, elevation 1362 m). Data from this station are available for the years 1961–2001. For the period 2001–2017 we used data from the Labská bouda station, which is located nearby (N50.77, E15.55, 3 km from Szrenica, elevation 1310 m). Data from Labská bouda were corrected using parameters of linear regression calculated over the period of simultaneous measurement for both stations (1979–1999). Correlations indicate that there was virtually no difference in temperature between those stations (*r* = 0.97–0.98 for individual months), and their precipitation was also highly significantly correlated (*r* = 0.61–0.89).

### Xylogenesis-Based Estimation of Growth Dynamics

First, the dates of the beginning/ending of EN phase were identified for each tree in terms of the first/last day for which at least two tracheid files contained enlarging tracheids (Rathgeber et al., 2018). Next, to describe the intra-annual progress of increasing number of cells forming a tree-ring, we first averaged the numbers of tracheids in different developmental phases from different files of a single tree ring and then calculated the sum of cells in enlarging, wall-thickening and mature phases (EWM) for each sample. To remove the effect of possible differences in growth speed around the stem, we multiplied number of EWM in each sample by the mean number of the previous year’s cells across all samples divided by the number of the previous year’s cells in that sample (Rossi et al., 2003). Although this standardization did not significantly modify intra-annual patterns of increasing tracheid number (Supplementary Figure S4), we chose to perform this statistical treatment due to the high eccentricity of some tree-rings and associated differences in tracheid numbers around the stem circumference (Treml et al., 2015). The number of EWM cells for each sampling date was divided by the final number of EWM cells at the end of the growing season to obtain for each day the percentage of the annual cell number. All analyses of xylogenesis data were performed using the “CAVIAR” package (Rathgeber et al., 2018) in R (R Core Team, 2020).

### Modeling Intra-Annual Growth Dynamics

The Vaganov-Shashkin process-based model (Vaganov et al., 2006) was used to reconstruct daily growth dynamics in the treeline ecotone over the period 1961–2017. The VS-model is a purely climate-driven model of tree-ring formation and cambial activity applicable to conifers regardless of geographical distribution or environmental variables (Anchukaitis et al., 2006; Touchan et al., 2012; Yang et al., 2017; Jevšenak et al., 2020). Here we highlight the most important aspects of the model regarding the way it is used in this study; for a full description of the model, see Vaganov et al. (2006).

**FIGURE 2:**
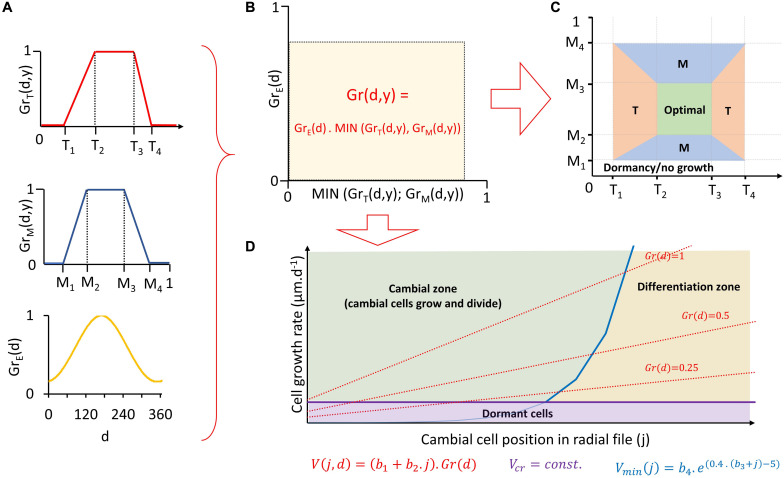
Visual representation of individual steps of Vaganov-Shashkin process-based model in environmental block **(A–C)** and cambial block **(D)**. Estimation of daily partial growth rates based on temperature, moisture, and daylength **(A)** and their conversion into daily integral growth rate **(B)**. Assessment of dominant climatic growth-limiting factor for each day **(C)** and calculation of position specific daily growth rates of cambial cells and assessment of their daily fate (growth and division, differentiation or dormancy) **(D)**. T = temperature; M = moisture; T_1__… 4_, M_1__… 4_ = threshold temperatures/moisture defining non-linear shape of partial growth rates response to climatic variable; Gr_T_(d,y), Gr_M_(d,y) = daily partial growth rates to temperature/moisture; Gr_E_(d) = daily daylength to daylength of summer solstice; Gr(d,y) = daily integral growth rate; V(j,d) = daily position-specific cell growth rate; V_*min*_(j) = growth rate determining transition from cambial zone to differentiation zone; b_1__–__4_, V_*cr*_ = constants of cambial block; d = day; y = year; j = position of cell in radial file.

The first part of VS-model algorithm (often called “environmental block”) converts daily climatic conditions into a set of dimensionless indexes defining daily climatic potential for tree growth. Its inputs include site tree-ring width chronology (to calibrate/verify model parameters), daily resolved climatic data (mean temperatures and precipitation totals) and daylength estimated from site latitude (Shishov et al., 2016; Anchukaitis et al., 2020). A key component of the environmental block is a pair of non-linear response functions that convert both temperature and soil moisture (calculated by incorporated water-balance model; Thornthwaite and Mather, 1955) of day (d) in year (y) into partial growth responses to temperature [Gr_T_(d,y)] and moisture [Gr_M_(d,y)]. Partial growth responses represent climatically driven growth potential on a scale from 0 to 1 (Figure 2A). The lower of them (following Liebig’s law of the minimum) is later scaled by the ratio of daylength to the daylength of summer solstice [Gr_E_(d)] to estimate a daily integral growth rate [Gr(d,y)] (Figure 2B). In addition, each growing season day can be classified based on values of partial growth rates as temperature-limited (Gr_T_(d,y) < Gr_M_(d,y)), moisture-limited (Gr_M_(d,y) < Gr_T_(d,y)) or climatically optimal (Gr_M_(d,y) = Gr_T_(d,y) = 1) (Figure 2C). Integral growth rates can be summed separately for days of different climatic limitation to estimate their contributions to total annual growth (Tumajer et al., 2017).

In the next segment (“cambial block”), the VS-model simulates cambial cell kinetics and differentiation of new tracheids aligned in a radial file (Vaganov et al., 2006). The only input here is the series of daily integral growth rates [Gr(d,y)] simulated by the environmental block. First, the model determines the period of the year when cambial cells are active (i.e., growing and dividing). This period is delimited using a “heat-sum” approach (*sensu* Delpierre et al., 2019) in terms of the first and the last day when cumulative temperature over a specific period (t_*beg*_) exceeds a certain threshold (T_*beg*_; for overview of all paremeters see Table 1). Next, for each day of the period with active cambium, cell-specific growth rates of individual cambial cells [V(j,d) in μm/d] are estimated based on their positions in the radial file (j) and Gr(d, y) (Figure 2D). From V(j,d) the three possible fates are determined for each of these cambial cells every day, such that it either: (i) stops growing (V(j,d) < V_*cr*_); (ii) grows in diameter in “cambial zone” (V(j,d) > V_*min*_(j)); or (iii) loses ability to divide and starts differentiation to become a xylem tracheid by entering the “enlargement zone” (V_*min*_(j) > V(j,d) > V_*cr*_). If the cambial cell diameter exceeds certain threshold (Y_M_), it enters the mitotic cycle and divides into two daughter cambial cells with equal diameter. It is important to note that the VS-model simulates only the process comprising cambial cell growth, division and occurrence of new differentiating cells (entrance into enlargement zone), not specific stages of tracheid differentiation and processes determining final tracheid anatomy (e.g., lumen enlargement, wall lignification). Consequently, it is not possible to determine daily numbers of cells in specific phases of differentiation (EN, WT, or MC), only the total number of cells that are in any of those stages (EWM). Simulated tree-ring width chronology is defined as the final annual simulated number of differentiated cells standardized by the mean number of differentiated cells per year over entire chronology period.

**TABLE 1:** List of VS-model parameters.

Parameter	Description	Value [unit]
**Environmental block**		
T_1_	Minimum temperature for growth	5 [°C]
T_2_	Lower threshold of optimal temperatures	11 [°C]
T_3_	Upper threshold of optimal temperatures	20 [°C]
T_4_	Maximum temperature for growth	30 [°C]
T_beg_	Cumulative temperature threshold to onset growth	46 [°C]
t_beg_	Length of window to calculate cumulative temperature	16 [day]
M_1_	Minimum soil moisture for growth	0.1 [v/v]
M_2_	Lower threshold of optimal soil moisture	0.6 [v/v]
M_3_	Upper threshold of optimal soil moisture	1.0 [v/v]
M_4_	Maximum soil moisture for growth	1.0 [v/v]
**Cambial block**		
V_cr_	Minimal cambial cell growth rate	0.04 [μm/day]
b_1_		0.25 [μm/day]
	Coefficients of position-specific cambial cell growth rate V(j,d) = (b_1_ + b_2_.j). Gr(d)	
b_2_		0.13 [μm/day]
b_3_		0.015 [μm/day]
	Coefficients of position-specific minimal cambial cell growth rate V_min_(j) = b_4_. (EXP(0.4*(b_3_ + j))-5)	
b_4_		2.5 [μm/day]
Y_G__1_	The maximum size of cambial cell in G1 phase	8 [μm]
Y_S_	The maximum size of cambial cell in S phase	9 [μm]
Y_G__2_	The maximum size of cambial cell in G2 phase	9.5 [μm]
Y_M_	The maximum size of cambial cell in M phase	10 [μm]
V_0_	The growth rate in the S, G2 and M phases	2 [μm/day]
t_c_	Time-step of cambial block	1 [day^–1^]
τ	Acclimation period of the cambial cell response to drop of temperature	3 [day]
**Soil moisture model (Thornthwaite and Mather, 1955)**		
M_0_	Initial soil moisture	0.6 [v/v]
l_r_	Rooting depth	300 [mm]
P_max_	Maximum precipitation for soil saturation	90 [mm]
C_1_	Proportion of precipitation not intercepted by canopy	0.6 [-]
C_2_	First coefficient of transpiration	0.0625 [mm/day]
C_3_	Second coefficient of transpiration	0.2 [°C^–1^]
Λ	Coefficient of water drainage from soil	0.01 [–]

*v/v–relative volumetric soil moisture content.*

Our initial exercise with original VS-model algorithm (Vaganov et al., 2006) indicated overall good performance of the model, but delays in cell formation simulations from observations in some years with cold and highly variable summer temperature. To cope with this problem, we modified original version of VS-model by implementing a simplified version of “acclimation of physiological processes to temperature” concept into cambial block (Gennaretti et al., 2017). Indeed, if temperature shortly drops below T_1_ threshold during the main growing season (delimited by DOYs 150–250), position-specific growth rates of cambial cells (V(j,d)) do not drop to 0 immediately, but only after acclimation period determined by the new parameter τ. In our case, τ = 3 days provided the best results when testing all possible values between 0 and 30 days. During the acclimation period, V(j,d) of all cambial cells equal V_*cr*_ (minimal growth rate before the cell enters dormancy). This means, that cambial cells can sustain radial growth and undergo mitosis but are not permitted to enter differentiation zone during acclimation period. Thus, the growth processes are active only in the cambial zone and new enlarging tracheids do not appear during acclimation.

Vaganov-Shashkin model is driven by a set of 10 parameters of the environmental block and 12 parameters of the cambial block; in addition, there are seven parameters of hydrological model simulating soil moisture. We used the standard methods to find or set their values (Shishov et al., 2016; Anchukaitis et al., 2020). First, we found optimal values of environmental block and hydrological model parameters by iterative sensitivity analysis (modifying values of each of the parameters and checking the response in coherency between the simulated and observed site chronologies). We compared simulated chronology primarily with standard chronology, but because of significant alteration of long-term tree-ring width trends by air pollution in 1980s in our study site (Kolář et al., 2015), we verified also the coherence between simulated and residual chronology. Because parameters of the cambial block were documented to be consistent across species and environments (Vaganov et al., 2006; Anchukaitis et al., 2020), we used their default values. As the final simulation, we retained the model that resulted in the lowest root-mean-square error (RMSE) between observed and simulated proportions of differentiating cells (EWM) for individual days of xylogenesis monitoring.

Independently, we verified estimated values of parameters by employing the differential evolution approach using a high-performance computer cluster. This approach finds values of a multidimensional domain of real-valued functions, which are optimal for a given environment (Price et al., 2005). All values estimated using the iterative approach occurred within the corresponding 95%-confidence limits yielded by differential evolution approach.

### Model Outputs, Validation of Its Performance and Simulated Cambial Dynamics

The following data were extracted from VS-model outputs: simulated tree-ring width chronology; cumulative annual growth rate during days with different climatic limitations; and simulated number of differentiating cells in radial file for each date of xylogenesis sampling, standardized by the final number of differentiated cells at the end of the year. In addition, we extracted four key dates describing the timing of cambial phenology and tree-ring formation for each year from the cambial block. As mentioned above, the VS-model estimates timing of cambial cell spring reactivation and autumn dormancy using the “heat-sum” approach (Yang et al., 2017; He et al., 2018b). We also obtained dates for the first and last appearance of differentiating cells in the radial file, i.e., the entrance of the first/last tracheid into the enlargement phase (Anchukaitis et al., 2020). Long-term trends in cambial phenology were quantified using linear regression. To assess trends in growth rates, we estimated the dominant climatic limiting factor (moisture limitation, temperature limitation, or optimal growth) based on specific values of Gr_M_ and Gr_T_ for each day (see section “Modelling Intra-annual Growth Dynamics” for details). Finally, we summed daily growth rates for dates with different types of climatic limitation and quantified their trends using linear regression.

The validation of the VS-model performance was based on a direct comparison of observed cambial dynamics with the respective outputs of simulations. Both observations and simulations provide numbers of EWM cells in a radial file for each day that xylogenesis sampling was done. To visualize intra-annual model performance, we overlaid plots of increasing numbers of simulated and observed EWM cells during 2010–2017. Statistical comparison of simulated and observed numbers of EWM cells was performed for each year using correlation coefficient (to assess similarity of intra-annual patterns) and Mann–Whitney *U* test (random dispersion of simulations around observations). In addition, the date of the first/last occurrence of enlarging cell (observation) is mimicked by the date of the first/last entrance of cell to differentiation zone (simulation). We constructed scatterplots between the simulated and observed dates of the first and last appearance of differentiating cell and calculated their regression parameters. Temporal stability of model parameters and their independence on the range of calibration data was tested using a bootstrapped transfer stability tests (Buras et al., 2017) between (i) simulated and observed chronologies and (ii) simulated and observed proportions of formed cells for each date of xylogenesis sampling. This test splits the datasets into two independent subperiods of equal length and compares bootstrapped confidence intervals of linear regression parameters between observation and simulation data for those subperiods.

## Results

### Site Chronology

Mean inter-series correlation and EPS (*sensu* Wigley et al., 1984) of the site chronology over the period 1961–2017 exceed 0.57 and 0.99, respectively. Standard chronology shows significantly (*p* < 0.001) increasing trend in tree-ring widths since 1961, steepening during the most recent decades (Supplementary Figure S5). Surprisingly, long-term increasing trend is significant also for residual chronology.

### Simulated Chronology and Model Parameters

**FIGURE 3:**
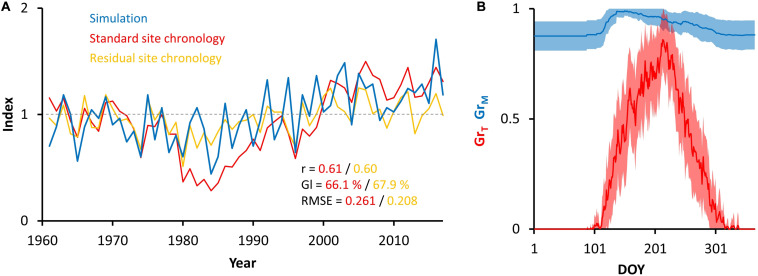
Annually resolved outputs of VS-model. Comparison of simulated (blue) and observed standard (red) and residual (yellow) chronologies together with their coherency statistics **(A)**, and intra-annual variability in mean (±SD) partial growth rates related to temperature (red) and soil moisture (blue) **(B)**. r = Pearson correlation coefficient; Gl = Synchronicity index; RMSE = root mean-squared error.

The correlation coefficients of simulated chronology with observed standard and residual chronologies are 0.61 and 0.60, respectively (Figure 3A). The simulated chronology high-frequency variability is synchronized with high-frequency variability of both site chronologies. Moreover, the trend of simulated chronology is coupled to trend of standard site chronology over all the periods without the influence of air pollution (1960–1980, 1990–2017). The final set of VS-model parameters is shown in Table 1. During iterative parameterization we observed the highest sensitivity of simulated chronology to variation in parameters related to effect of low temperature (T_1_, T_2_) and high soil moisture (M_3_, lr, Λ) on tree growth (Supplementary Figure S6). Interestingly, the excessive soil moisture does not act as a growth-limiting factor in our study region, as the VS-model provided significant coherence between simulated and observed chronology only in the case of M_3_ = M_4_ = 1. According to the bootstrapped transfer stability tests, the coherence between simulated and observed chronology is stable over time (Supplementary Table S4).

### Simulated Intra-Annual Growth Rates

On average, the growth at our study region was limited by low temperature during entire growing season (Figure 3B). The mean daily partial growth response to moisture (Gr_M_) did not drop below 0.87, whereas the partial growth response to temperature (Gr_T_) peaked at 0.86 (DOY 213). The highest growth rate occurred between DOY 172 and 239, when mean Gr_T_ exceeds 0.6.

**FIGURE 4:**
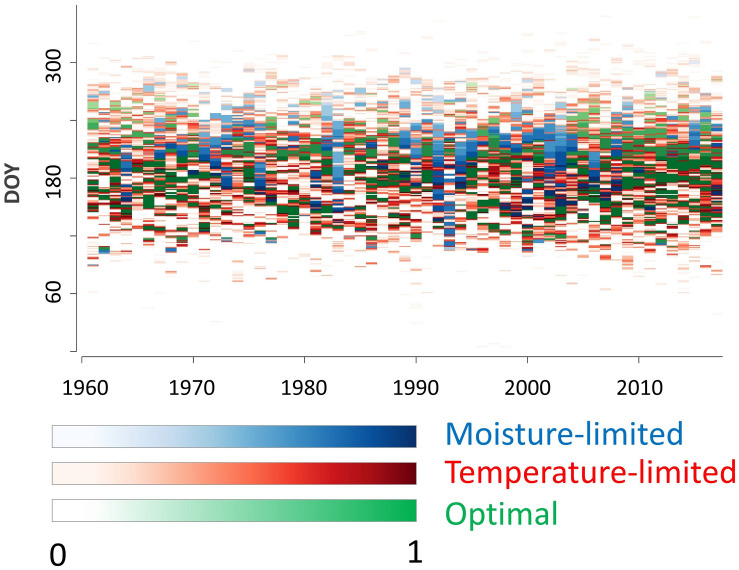
Simulated daily integral growth rates. Color scale indicates the value of integral growth rate and color refers to the dominant climatic limiting factor.

Considering daily model outputs, however, periods of various durations (from single days to months) with drought limitation or climatically optimal growth were identified in specific years. In total, 10773 days of cambial activity were simulated for the period 1961–2017 (Figure 4). Temperature was the principal growth-limiting factor controlling on average 43% of tree-ring formation (i.e., cumulative Gr during days with Gr_T_ < Gr_M_), whereas drought dominated in 22% (Gr_M_ < Gr_T_). The remaining 35% of growth happened during optimal climatic conditions (Gr_M_ = Gr_T_ = 1).

**FIGURE 5:**
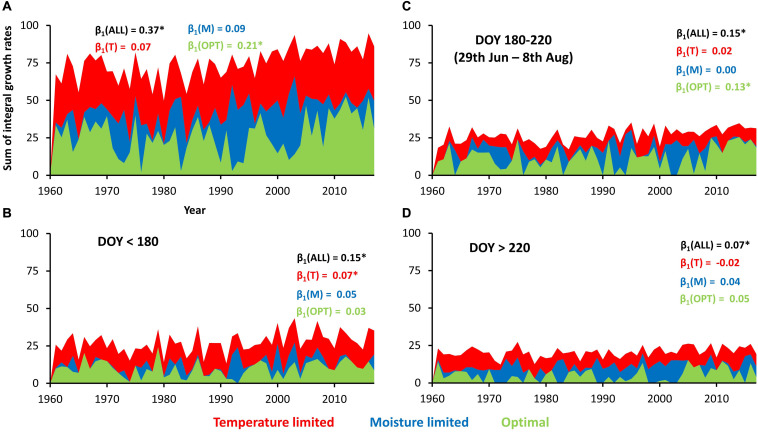
Sum of integral growth rates under different types of climatic limitation over entire year **(A)** or specific parts of growing season **(B–D)**. β_1_ indicate slopes of linear trends of integral growth rates summed during entire year (black) and during days with specific climatic limitations (colors). Asterisk indicates significant slope (*p*-value < 0.05).

On average, annual tree rings consisted of 49.8 simulated cells during 1961–2017. There was a significant positive trend in simulated number of cells forming a tree-ring for 0.42 cell year^–1^ (≈0.8% of long-term mean). Similarly, total sum of integral growth rates over the entire growing season increased by 0.37 year^–1^ (≈0.5% of long-term mean), which was driven mainly by increasing growth in climatically optimal conditions (0.21 year^–1^; Figure 5A). If the growing season is split into three segments each contributing similar proportion to total tree-ring width (before DOY 180, 180–220, and after 220), the significant positive trends for total growth are found in all of them. The highest increases of simulated growth rates were documented during spring (increasing growth under temperature limitation; Figure 5B) and summer (increasing growth under climatically optimal conditions; Figure 5C), while autumn positive trend is weaker (Figure 5D).

### Simulated Growth Phenology and Progress of Tree-Ring Formation

**FIGURE 6:**
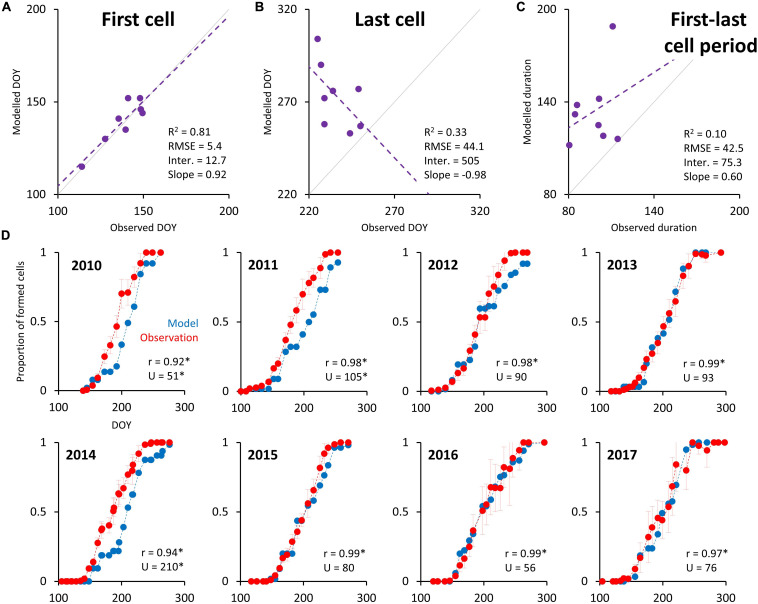
Scatterplots between simulated and observed dates of the appearance of the first **(A)** and the last **(B)** differentiating cell in the radial file, and between simulated and observed durations of the period between the first and the last appearance of enlarging cell **(C)**. Intra-annual progress of increasing number of simulated and observed differentiating cells **(D)**. Gray lines in panels **(A–C)** indicate 0–1 relationship and dashed lines indicate least-square regression with parameters shown in the bottom left corners. Error bars around observation points in panel **(D)** represent 95% confidence intervals. r and U in panel **(D)** indicate correlation coefficient and statistics of Mann–Whitney *U* test between both lines, respectively. Asterisk indicates statistically significant statistics (*p*-value < 0.05).

The mean residual in the estimated date of the occurrence of the first enlarging tracheid is 4.5 days (Figure 6A), which is less than the mean xylogenesis sampling frequency of 8 days. Unlike this high degree of accuracy for predicting growing season activity onset, the model’s predicted dates for the last differentiating cell are on average 37.5 days later than those observed (Figure 6B). Consequently, the simulated duration of the period between first and last enlarging cell appearance is on average 36 days longer than in case of observation (Figure 6C). The intra-annual pattern of increasing number of EWM cells highly significantly correlates with observation data for all years (*r* > 0.92), and residuals are randomly distributed according to Mann–Whitney *U* test for all years except 2010, 2011, and 2014 (Figure 6D). For those years the model simulated delayed tree-ring formation mainly in the middle part of the growing seasons (DOY 170–230). The regression between simulated and observed progress of increasing cell number is temporary stable in terms of mean (intercept) and coherence (R^2^), but the similarity of variability (slope) slightly increases over time (for 6% between the first and second half of the dataset; Supplementary Table S4).

**FIGURE 7:**
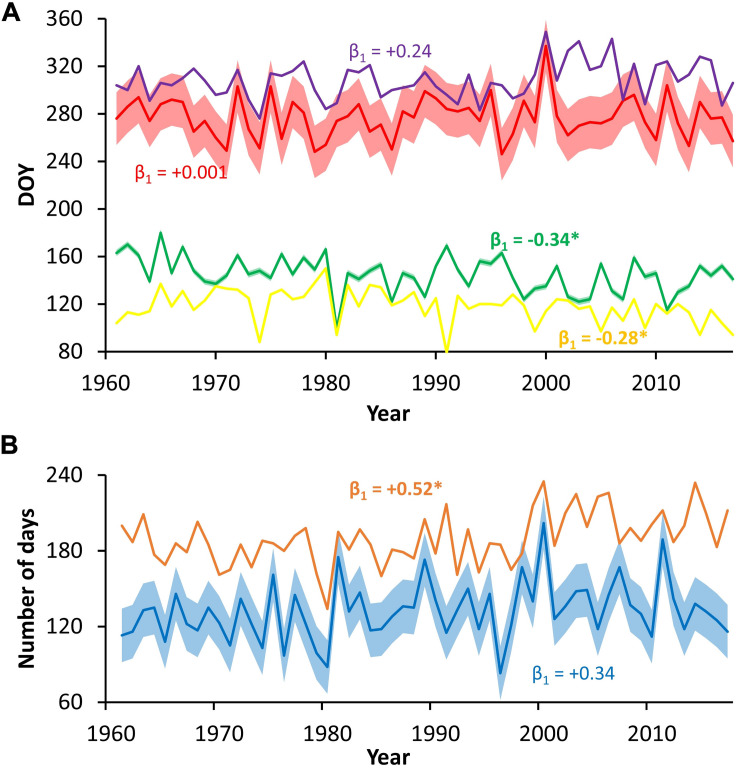
Time series of simulated cambial phenology phases timing **(A)** and duration **(B)**; colors represent the following: yellow–onset of cambial cell growth, green–appearance of the first differentiating cell, red–appearance of the last differentiating cell, purple–cessation of cambial cell growth, blue–period between appearance of the first/last differentiating cell, brown–period between onset/cessation of cambial cell growth; error belts around series of the first/last occurrence of differentiating cell and period between them indicate RMSE of estimates; β_1_ = slope of linear regression, significant slopes are given in bold with asterisk.

All simulated time series of growth phenology showed prominent year-to-year variability and less pronounced long-term trends. On average, the onset of cambial cell growth, first differentiating cell, last differentiating cell, and cessation of cambial cell growth occurred at DOY 118 (±14), 145 (±15), 277 (±17), and 308 (±15), respectively. There were negative temporal trends in cambial cell growth onset and appearance of the first differentiating tracheid, and positive trends in autumn cambial cell growth cessation and appearance of the last differentiating tracheid (Figure 7A). However, only the trends in the onset of cambial cell growth (0.28 d/y) and the first differentiating tracheid appearance (0.34 d/y) were significant based on linear regression. Trends in the growing season duration are influenced by unprecise estimate of the growing season cessation (Figure 7B).

## Discussion

The results of this study demonstrate that combination of process-based models of wood formation and extensive observational data allows the identification of specific model limitations as well as extension of our understanding of processes modulating cambial activity on long temporal scales. Below, we frame implications of our results relevant for both methodology (limitations of the VS-model indicating the future development directions) and environment-growth interaction (climate-change effect on cambial dynamics at the treeline).

### Capabilities and Potential Application of the VS-Model

The VS-model precisely predicted the date of the first differentiating tracheid appearance, for which mean model residuals are smaller than the frequency of xylogenesis sampling. Moreover, simulated and observed proportions of formed cells are tightly synchronized mainly at the beginning of the tree-ring (Figure 6). This is not surprising, because onset of cambial activity (Rossi et al., 2008, 2016; Treml et al., 2015; Delpierre et al., 2019; Huang et al., 2020) and regulation of first phases of xylogenesis (Deslauriers and Morin, 2005) are strictly temperature-controlled in cold environments. The algorithm of the VS-model uses the cumulative temperature threshold to estimate the date of spring onset of cambial cell growth and, in the next step, calculates daily resolved kinetics of cell growth and differentiation based on the cell position in the radial file and climatically driven external growth rates (Vaganov et al., 2006). The VS-model assumes a non-linear response between rate of cambial processes and climatic conditions during the growing season, which is a more realistic shape of the response function than the frequently used linear regression (Lloyd et al., 2013; Jevšenak and Levanič, 2016; Sun et al., 2017; Gu et al., 2019). Consequently, simulations of the VS-model–as a purely climate-driven process-based model–correspond to observation data especially in periods with dominant climatic control on cambial growth.

Indeed, VS-model does not consider any of non-climatic factors which might influence process of cell division and differentiation. The simulated progress of increasing number of cells was synchronized with xylogenesis data for five of eight considered years, while for other 3 years the model suggested delayed formation of the tree-ring. Consequently, simulated dates of the last differentiated cell appearance are unprecise. The termination of xylem formation is regulated partly by internal physiological processes, for instance, intra-annual patterns of hormone concentrations (Hartmann et al., 2017) or filling non-structural carbohydrate storage pools (Furze et al., 2019). The end of the maturation phase often occurs before climatic conditions deteriorate to levels expected to limit cambial growth (Friend et al., 2019). Moreover, ending of cambial activity usually shows the highest level of variability among trees of all xylem developmental phases (Deslauriers et al., 2008; Lupi et al., 2010; Rossi et al., 2016), suggesting existence of individual-tree regulation not considered by the VS-model.

The declining precision of VS-model through growing season resulting in biased estimate of growing season cessation was recently confirmed also in a boreal cold forests in Canada (Buttò et al., 2020). Surprisingly, while Buttò et al. (2020) observed systematic delay of simulated cambial kinetics from the first cells of the radial file, in our case, simulation was coherent with observations during entire growing season in 5 of 8 years. In three remaining years, simulations were still tightly coupled with observations for approx. first 25% of tree-ring. We suggest that simulated intra-annual growth dynamics was in this study significantly improved by implementation of “temperature acclimation principle” into cambial block. Cell division does not stop immediately in response to cooling (Gričar et al., 2006). Indeed, limited but slowly ceasing growth is sustained between 0 and 5°C (Körner, 2003; Rossi et al., 2007), and this mechanism was not considered by previous version of the VS-model (Vaganov et al., 2006). In addition, we used original process-based approach to simulate cambial dynamics implemented in the cambial block of the VS-model, while the work of Buttò et al. (2020) determined cambial dynamics using statistical links between cumulative integral growth rates and relative cell position (Popkova et al., 2018). Higher precision of our simulations confirms that statistical approaches might not represent appropriate solution for modeling cambial dynamics over long temporal scales (Friend et al., 2019).

### Parameters of the VS-Model

The set of estimated VS-model parameters is realistic in terms of physiological thresholds of *P. abies* and environmental conditions at our study site. The sensitivity analysis based on parameters of environmental block revealed, that variability in tree-ring widths is driven primarily by the effect of low temperatures and high soil moisture, which are common growth determinants at the treeline environment (Körner, 2012). The estimated minimum temperature to sustain cambial cell enlarging (T_1_ = 5°C) is similar to values reported for most treeline coniferous species based on xylogenesis observations (approx. 5–9°C; Rossi et al., 2007). In addition, cumulative temperature to onset cambial cells growth (T_*beg*_/t_*beg*_ = 46°C per 16 days) yielded mean date of cambial reactivation at the end of April or beginning of May, which agrees with local observations (Treml et al., 2015). Regarding soil moisture parameters, it is worth mentioning, that more than 90% of fine root biomass of *P. abies* was documented to occur at a depth of less than 0.3 m on acidic soils (parameter lr; Schmid and Kazda, 2002; Puhe, 2003). Additionally, the parameters of the hydrological component of the VS-model–namely low level of canopy interception (C_1_ = 60% of non-intercepted precipitation) and very high soil water drainage–are also realistic for treeline with low canopy cover and steep slopes. Although the climate of the study region is cold and rainy, the growth of trees is not limited by high soil moisture content (Kolář et al., 2015; Ponocná et al., 2016). This is probably because of extremely fast soil water drainage and rocky soils with a high level of aeration. Consequently, the VS-model flexibly adjusted a response function to moisture by omitting a segment with inverse response between soil moisture and growth (i.e., M_3_ = M_4_ = 1).

### Trends in Simulated Growth Phenology and Kinetics

The only simulated phenological phases with significant trends are the dates of cambial cell growth onset (0.28 d/y) and the first occurrence of a differentiating cell (0.34 d/y). Those values are noteworthy higher than estimates based purely on climatic data from our study region (0.001 d/y; Dubicka and Głowicki, 2000). Climate-based estimates of growth phenology suffer from oversimplification of climate-growth relationships by not taking cambial processes into account (Cuny et al., 2014; Friend et al., 2019). The VS-model, in contrast, does not predict key dates of cambial activity based purely on simple climatic thresholds, but by explicitly modeling processes of cambial cell enlargement, mitosis and tracheid differentiation. Consequently, the simulated trends in spring phenological phases are closer to values reported from other mountainous regions similar to the Krkonoše Mts. where they were estimated using empirical or modeling approaches, e.g., the Alps (0.20 d/y; Prislan et al., 2019) and the Tibetan plateau (0.28 d/y; Yang et al., 2017).

Both simulated numbers of cells and annual sum of integral growth rates show significant positive trends. Their slopes span between 0.5 and 0.8% year^–1^, which are similar values to linear trend inferred from standard chronology (0.8% year^–1^). Our study site–similarly to the majority of treelines in the Central Europe (Büntgen et al., 2006; Ponocná et al., 2016; Wieser et al., 2019)–shows an increasing trend in tree-ring width since 1960s, which is especially prominent during the last three decades. Intra-annual model outputs reveal that this trend is driven by cambial kinetics stimulation mainly in the early (DOY < 180) and peak (DOY 180–220) parts of growing season, while the contribution of the late part (DOY > 220) of growing season is marginal. The climatic drivers underlying increasing growth rates differ between the early and peak part of growing season. In spring increasing temperatures stimulate higher growth rates during temperature-limited days (T_1_ < T < T_2_). By contrast, temperature increase in summer results in more frequent occurrence of climatically optimal days with growth rates decoupled from the course of temperature (T_2_ < T < T_3_). This coupling/decoupling of spring/summer growth rates from the climatic conditions might have a significant consequences for the temporal stability of the climatic signal stored in tree-ring width chronologies (D’Arrigo et al., 2008).

## Conclusion

Comparison of simulated intra-annual growth dynamics with xylogenesis observations revealed high model precision in estimating the day of the tracheid differentiation onset. Similarly, the model exhibits high validity in simulated annual number of tracheids produced by cambium (i.e., tree-ring width) and intra-annual cambial dynamics (i.e., progress of increasing cell number during the year). By contrast, the simulated date of the last enlarging cell appearance is biased and together with the simulated growing season duration should be interpreted with caution. Challenging future task is to improve the VS-model performance in the late part of growing season while keeping this model widely applicable without the need for rarely available physiological input data.

Using the process-based climate-driven VS-model, we showed that the pronounced growth increase recently observed in treeline stands of Central Europe results mainly from increased growth rates during early and peak growing season. Summer growth rates follow increasing trends of temperatures and become substantially less temperature-limited. In addition, earlier onset of cambial activity and higher spring temperatures promote increasing spring growth rates, which, however, remain mostly temperature-limited. Given the marginal importance of moisture availability as growth-limiting factor, the further increase of intra-annual growth rates and annual tree-ring widths might be anticipated at treeline with ongoing climate change.

## Data Availability Statement

The raw data supporting the conclusions of this article will be made available by the authors, without undue reservation.

## Author Contributions

JT developed the idea of the study, analyzed the data (with contribution of VS, IT, MP, and VT), and led writing of the manuscript. VT, HK, and JK performed the fieldwork. All authors contributed to writing of the manuscript and approved its final version.

## Conflict of Interest

The authors declare that the research was conducted in the absence of any commercial or financial relationships that could be construed as a potential conflict of interest.

## Footnotes

1 https://www6.nancy.inrae.fr/foret-bois-lerfob/Projets/Projets-en-cours/GLOBOXYLO (last accessed 16.11.2020).
