# Supplementary material for: Forward Modeling Reveals Multidecadal Trends in Cambial Kinetics and Phenology at Treeline
Source: Front Plant Sci. 2021 Jan 28;12:613643. doi: 10.3389/fpls.2021.613643 (PMC7875878; doi:10.3389/fpls.2021.613643)
Supplement: Supplementary file 1 [file Image_1.PDF]

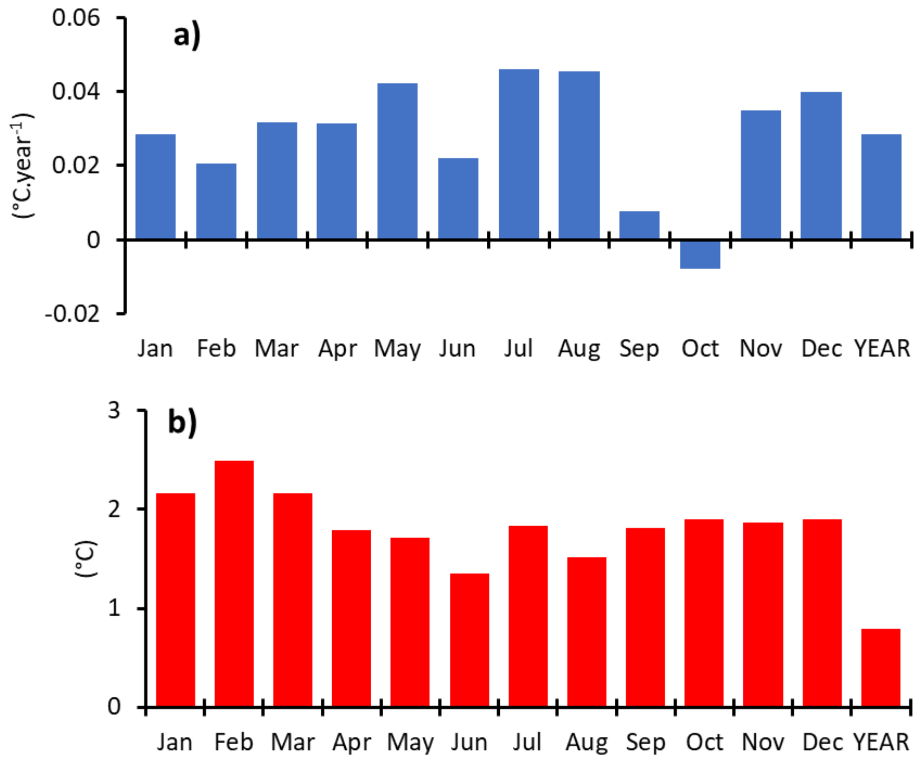

Figure S1: Slopes of linear trends (a) and standard deviations (b) in monthly and annual mean temperatures over period 1961-2017
