# Supplementary material for: Forward Modeling Reveals Multidecadal Trends in Cambial Kinetics and Phenology at Treeline
Source: Front Plant Sci. 2021 Jan 28;12:613643. doi: 10.3389/fpls.2021.613643 (PMC7875878; doi:10.3389/fpls.2021.613643)
Supplement: Supplementary file 2 [file Image_2.PDF]

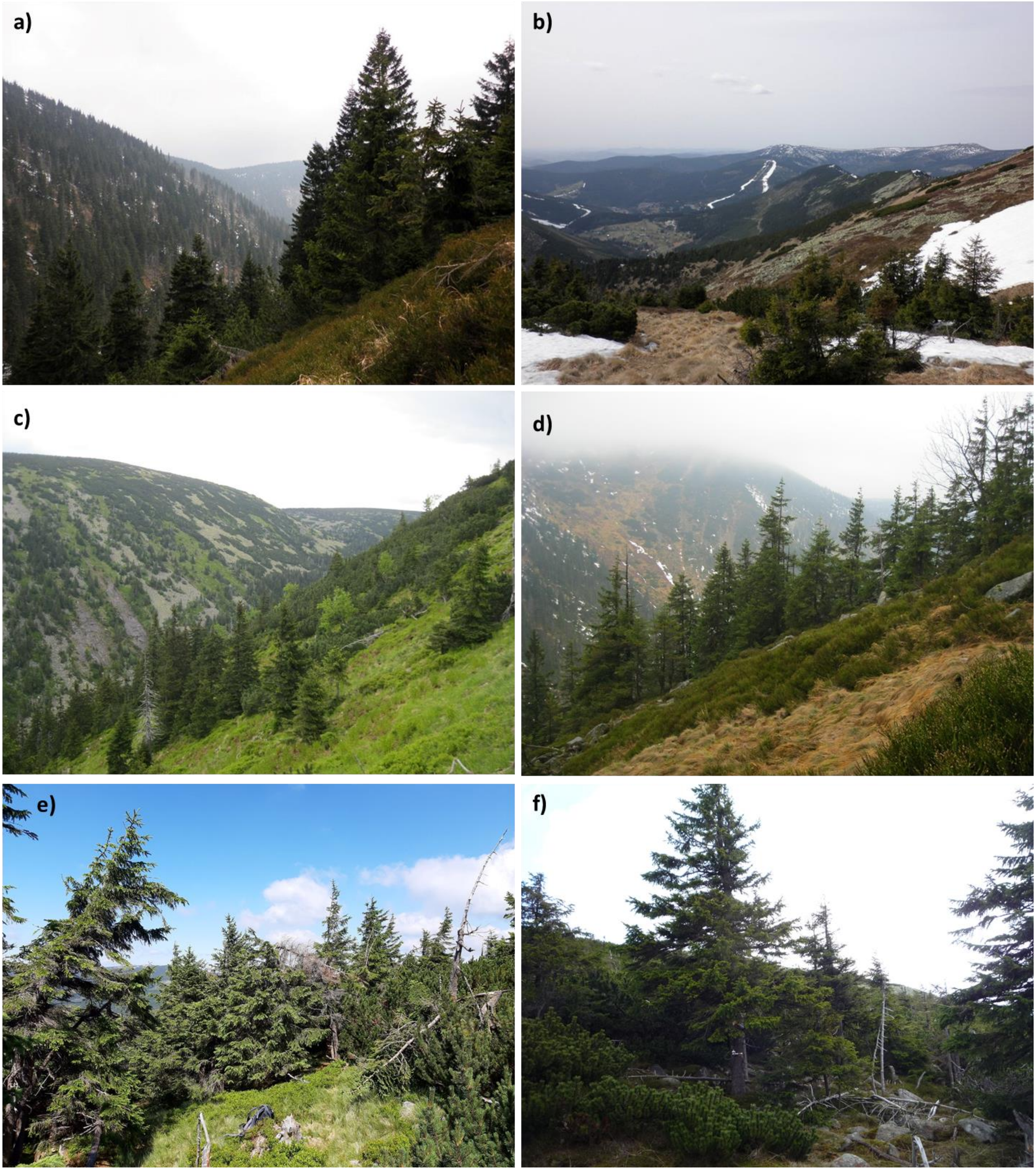

Figure S2: Pictures of xylogenesis sampling sites: Lucni hora – Timberline (a), Lucni hora – Treline (b), Bile Labe – North (c), Bile Labe – South (d), Maly Sisak – West (e) and Maly Sisak – East (f)
