# Supplementary material for: Forward Modeling Reveals Multidecadal Trends in Cambial Kinetics and Phenology at Treeline
Source: Front Plant Sci. 2021 Jan 28;12:613643. doi: 10.3389/fpls.2021.613643 (PMC7875878; doi:10.3389/fpls.2021.613643)
Supplement: Supplementary file 3 [file Image_3.PDF]

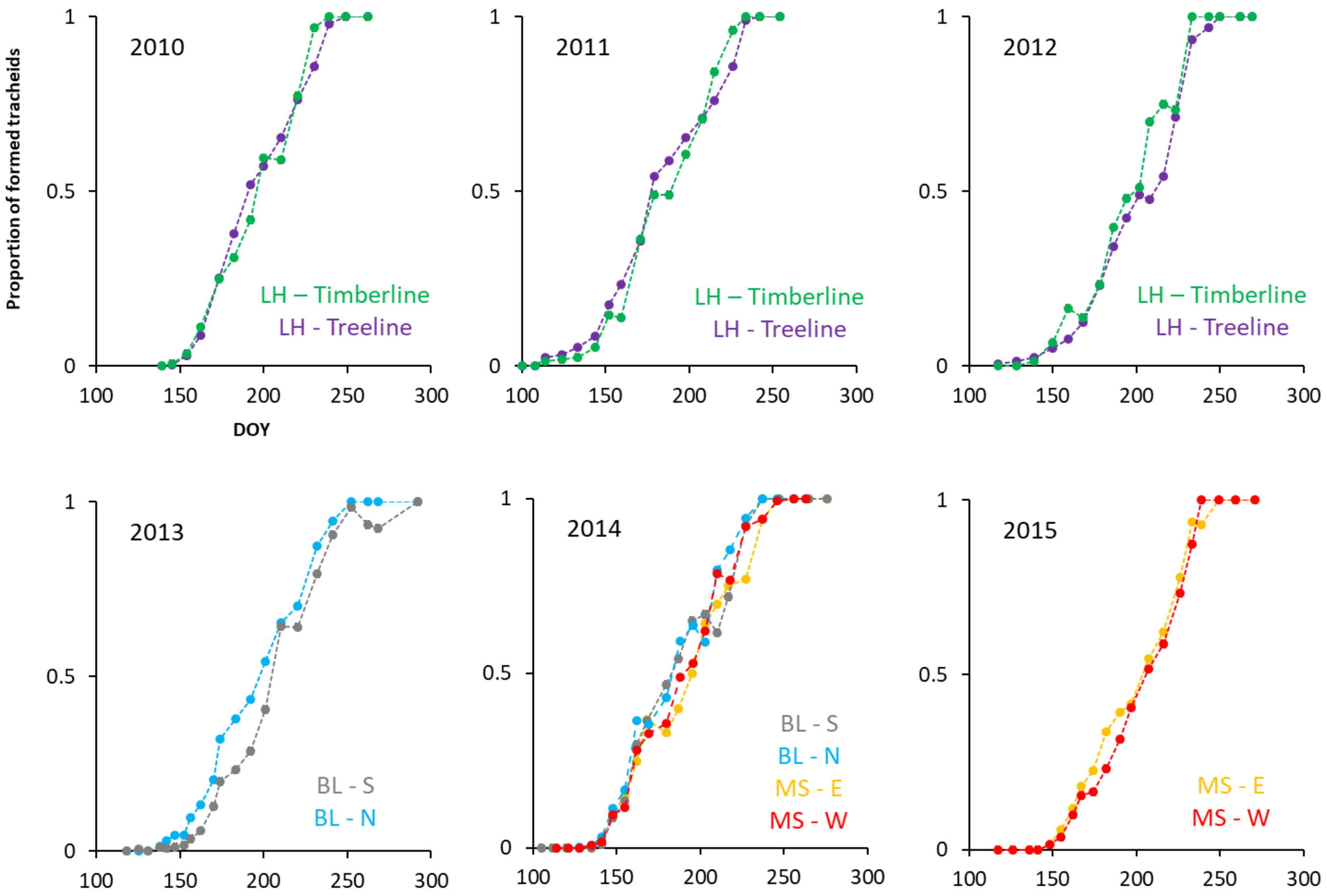

Figure S3: Comparison of cambial kinetics between individual sampling microsites. Graphs show the increasing proportion of differentiating tracheids (enlarging+wall-thickening+mature) for individual sampling dates standardized by the final number of cells formed at the end of the calendar year. Differences between microsites are non-significant for all sampling dates (n=165)
