# Supplementary material for: Forward Modeling Reveals Multidecadal Trends in Cambial Kinetics and Phenology at Treeline
Source: Front Plant Sci. 2021 Jan 28;12:613643. doi: 10.3389/fpls.2021.613643 (PMC7875878; doi:10.3389/fpls.2021.613643)
Supplement: Supplementary file 4 [file Image_4.PDF]

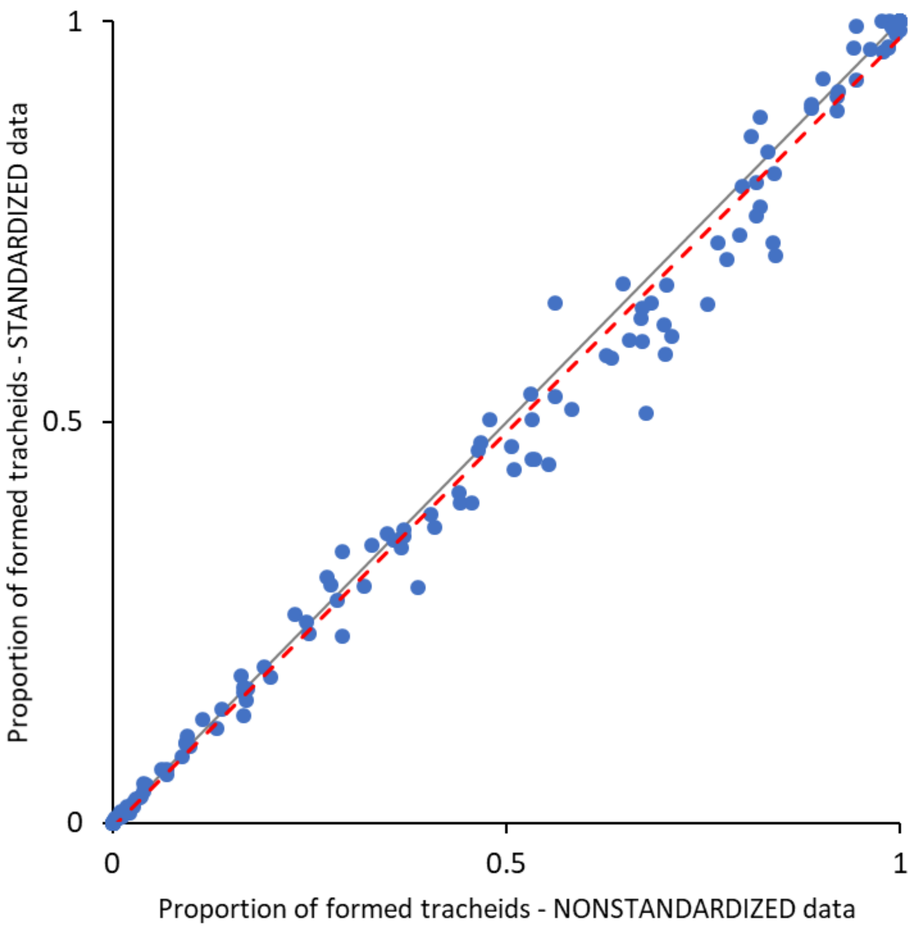

Figure S4: Relationship between proportion of tracheids in enlarging, wall-thickening and mature phases formed during individual dates of xylogenesis monitoring based on standardized and nonstandardized data. Red dashed line represents least-square regression.
