# Supplementary material for: Forward Modeling Reveals Multidecadal Trends in Cambial Kinetics and Phenology at Treeline
Source: Front Plant Sci. 2021 Jan 28;12:613643. doi: 10.3389/fpls.2021.613643 (PMC7875878; doi:10.3389/fpls.2021.613643)
Supplement: Supplementary file 5 [file Image_5.PDF]

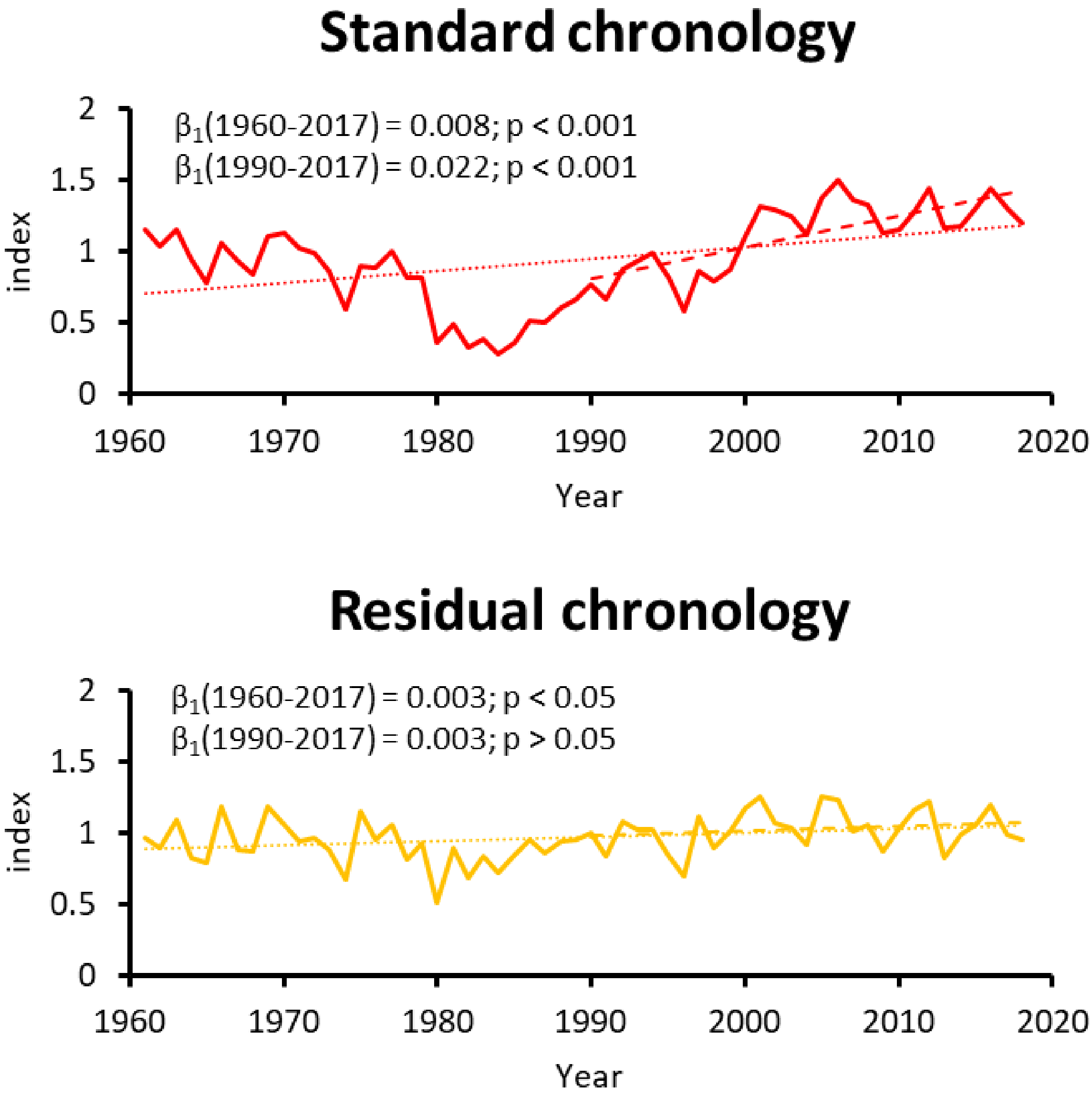

Figure S5: Multidecadal trends in site chronologies. Lines represent least-square regression for period 1960-2017 (dotted) and 1990-2017 (dashed). β_1 indicates slopes of those regressions
