# Supplementary material for: Forward Modeling Reveals Multidecadal Trends in Cambial Kinetics and Phenology at Treeline
Source: Front Plant Sci. 2021 Jan 28;12:613643. doi: 10.3389/fpls.2021.613643 (PMC7875878; doi:10.3389/fpls.2021.613643)
Supplement: Supplementary file 6 [file Image_6.PDF]

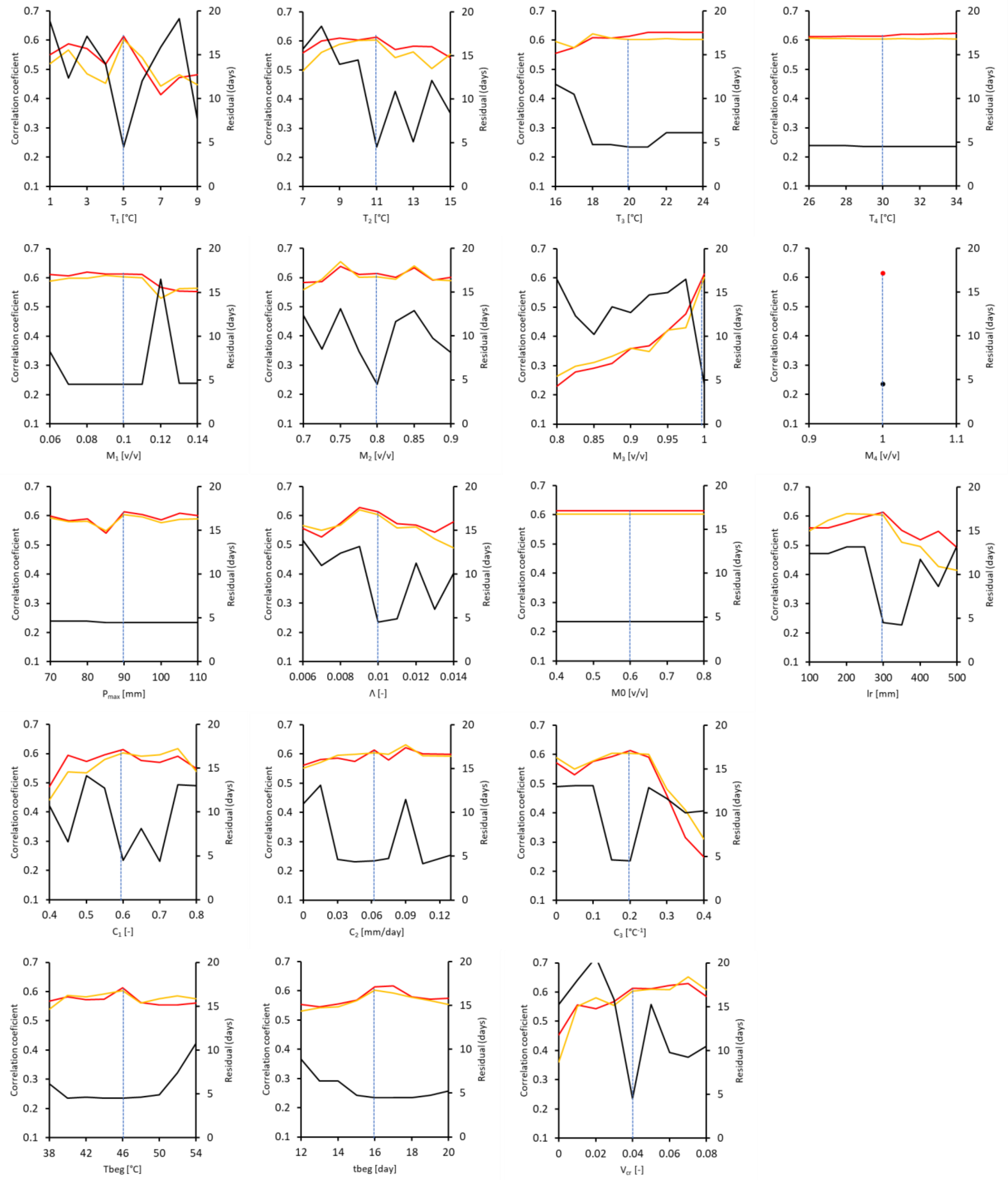

Figure S6: Sensitivity analysis of coherence between simulated chronology and standard (red) and residual chronologies (orange) and in estimates of the start of the growing season (black). Each chart shows response of correlation coefficient between simulated and observed chronologies and mean residual of estimated date of growing season onset to variation of specific parameter, while other parameters remained fixed. Only logical values of parameters were tested (e.g., $M_4 \geq M_3$). Blue vertical lines depict the finally selected parameter values.
