# Supplementary material for: Forward Modeling Reveals Multidecadal Trends in Cambial Kinetics and Phenology at Treeline
Source: Front Plant Sci. 2021 Jan 28;12:613643. doi: 10.3389/fpls.2021.613643 (PMC7875878; doi:10.3389/fpls.2021.613643)
Supplement: Supplementary file 7 [file Table_1.DOCX]

**Table S1**: Sites and sample depths of xylogenesis monitoring in individual years

| **Treeline segment** | **Microsite** | **Coordinates** | **Elevation (m a. s. l.)** | **Number of sampled trees** | | | | | | | |
| --- | --- | --- | --- | --- | --- | --- | --- | --- | --- | --- | --- |
|  |  |  |  | **2010** | **2011** | **2012** | **2013** | **2014** | **2015** | **2016** | **2017** |
| Lucni hora | Timberline | N50° 43.32’  E15° 40.32’ | 1310 | 8 | 9 | 6 |  |  |  |  |  |
|  | Treeline | N50° 43.45’  E15° 40.70’ | 1450 | 8 | 9 | 6 |  |  |  |  |  |
| Bile Labe | North | N50° 44.10’  E15° 39.54’ | 1250 |  |  |  | 8 | 8 |  |  |  |
|  | South | N50° 44.36’  E15° 39.97’ | 1250 |  |  |  | 8 | 8 |  |  |  |
| Maly Sisak | East | N50° 45.32’  E15° 39.06’ | 1310 |  |  |  |  | 8 | 8 |  |  |
|  | West | N50° 45.55’  E15° 38.73’ | 1370 |  |  |  |  | 8 | 8 | 6 | 6 |
