# Supplementary material for: Forward Modeling Reveals Multidecadal Trends in Cambial Kinetics and Phenology at Treeline
Source: Front Plant Sci. 2021 Jan 28;12:613643. doi: 10.3389/fpls.2021.613643 (PMC7875878; doi:10.3389/fpls.2021.613643)
Supplement: Supplementary file 8 [file Table_2.DOCX]

**Table S2**: Mean ± SD of dendrometric parameters of individual trees sampled for xylogenesis monitoring

| **Year** | **Region** | **Microsite** | **Age**  **[year]** | **Diameter at breast height [cm]** | **Height**  **[m]** |
| --- | --- | --- | --- | --- | --- |
| 2010 | Lucni hora | Timberline | 58 ± 7 | NA | 7.81 ± 0.87 |
|  |  | Treeline | 52 ± 10 | NA | 3.61 ± 0.51 |
| 2011 | Lucni hora | Timberline | 68 ± 23 | NA | 7.51 ± 0.68 |
|  |  | Treeline | 78 ± 28 | NA | 3.52 ± 0.47 |
| 2012 | Lucni hora | Timberline | 93 ± 30 | NA | 9.91 ± 0.84 |
|  |  | Treeline | 89 ± 22 | NA | 4.21 ± 0.41 |
| 2013 | Bile Labe | North | NA | NA | NA |
|  |  | South | NA | NA | NA |
| 2014 | Bile Labe | North | NA | 46 ± 18 | 9.19 ± 1.17 |
|  |  | South | NA | 36 ± 3 | 8.50 ± 0.50 |
|  | Maly Sisak | East | 77 ± 15 | 18 ± 2 | 5.76 ± 0.63 |
|  |  | West | 83 ± 11 | 20 ± 4 | 6.63 ± 0.60 |
| 2015 | Maly Sisak | East | 107 ± 29 | 20 ± 6 | 6.13 ± 0.97 |
|  |  | West | 91 ± 17 | 20 ± 6 | 6.33 ± 1.44 |
| 2016 | Maly Sisak | West | 87 ± 18 | 22 ± 6 | 6.40 ± 1.60 |
| 2017 | Maly Sisak | West | 72 ± 16 | 54 ± 9 | 5.95 ± 0.48 |
