# Supplementary material for: Forward Modeling Reveals Multidecadal Trends in Cambial Kinetics and Phenology at Treeline
Source: Front Plant Sci. 2021 Jan 28;12:613643. doi: 10.3389/fpls.2021.613643 (PMC7875878; doi:10.3389/fpls.2021.613643)
Supplement: Supplementary file 9 [file Table_3.DOCX]

**Table S3**: Pairwise comparison between mean estimated key dates of cambial activity dynamics for microsites (treeline x timberline, aspect) and treeline regions. Key dates were calculated using standard approaches implemented in CAVIAR package (*Rathgeber et al. 2018: Tree Physiology 38, 1246-1260*). P-values were estimated using unilateral bootstrap permutation test.

| **Year** | **Region** | **Microsite** | **bE** | | **bW** | | **bM** | | **cE** | | **cW** | |
| --- | --- | --- | --- | --- | --- | --- | --- | --- | --- | --- | --- | --- |
|  |  |  | **DOY** | **p-value** | **DOY** | **p-value** | **DOY** | **p-value** | **DOY** | **p-value** | **DOY** | **p-value** |
| 2010 | LUC | Timb | 147.8 | ns. | 166.6 | ns. | 181.4 | <0.01 | 232.8 | ns. | 255.1 | <0.01 |
|  |  | Tree | 149.1 |  | 164.8 |  | 179.4 |  | 225.4 |  | 236.0 |  |
| 2011 | LUC | Timb | 111.6 | ns. | 152.9 | ns. | 166.9 | <0.01 | 226.8 | ns. | 234.3 | ns. |
|  |  | Tree | 114.0 |  | 156.8 |  | 172.6 |  | 221.0 |  | 231.6 |  |
| 2012 | LUC | Timb | 120.8 | ns. | 163.7 | ns. | 175.3 | 0.02 | 234.5 | ns. | 248.7 | 0.03 |
|  |  | Tree | 134.3 |  | 159.2 |  | 169.8 |  | 222.5 |  | 239.3 |  |
| 2013 | BL | North | 137.6 | ns. | 165.9 | ns. | 181.9 | <0.01 | 239.1 | ns. | 276.6 | na |
|  |  | South | 141.6 |  | 173.4 |  | 192.3 |  | 251.6 |  | na |  |
| 2014 | BL | North | 139.9 | ns. | 156.1 | ns. | 171.3 | <0.01 | 231.4 | ns. | 259.8 | <0.01 |
|  |  | South | 143.3 |  | 158.9 |  | 179.9 |  | 225.8 |  | 249.4 |  |
| 2014 | MS | East | 139.4 | ns. | 154.7 | ns. | 168.0 | <0.01 | 222.1 | ns. | 249.1 | ns. |
|  |  | West | 141.6 |  | 154.3 |  | 165.9 |  | 230.1 |  | 248.5 |  |
| 2015 | MS | East | 147.5 | 0.04 | 162.8 | ns. | 178.6 | <0.01 | 237.6 | ns. | 259.5 | 0.03 |
|  |  | West | 151.5 |  | 165.5 |  | 181.5 |  | 231.5 |  | 253.4 |  |
| 2014 | BL | N+S | 140.8 | ns. | 158.1 | ns. | 175.5 | <0.01 | 228.4 | ns. | 255.3 | ns. |
|  | MS | E+W | 140.0 |  | 154.9 |  | 167.3 |  | 225.3 |  | 248.0 |  |
| 2012 | BL* | N+S | 134.0 | ns. | na | | | | | | | |
|  | LUC | Timb+Tree | 128.8 |  |  |  |  |  |  |  |  |  |

bE…beginning of enlarging; bW…beginning of wall-thickening; bM…beginning of maturation; cE…cessation of enlarging; cW…cessation of wall-thickening

* Sampling in 2012 in the Bile Labe valley was performed only during the beginning of the growing season (DOY 117-150). It is used here only to document similarity between LUC and BL sites and not in the rest of analyses in the main body of the manuscript.
