# Supplementary material for: Forward Modeling Reveals Multidecadal Trends in Cambial Kinetics and Phenology at Treeline
Source: Front Plant Sci. 2021 Jan 28;12:613643. doi: 10.3389/fpls.2021.613643 (PMC7875878; doi:10.3389/fpls.2021.613643)
Supplement: Supplementary file 10 [file Table_4.DOCX]

**Table S4**: Results of bootstrapped transfer stability test (*Buras et al. 2017: Dendrochronologia 42: 56-62*) between observation data and model simulations. P-values were estimated using 1000 bootstraps

| **Simulated and observed series** | **N** | **BTFS parameter** | **Intercept** | **Slope** | **R^2^** |
| --- | --- | --- | --- | --- | --- |
| **Tree-ring width chronologies** | 57 | bootstrapped estimate | 0.624 | 1.021 | 0.655 |
|  |  | p-value | 0.380 | 0.942 | 0.490 |
| **Simulated and observed proportions of differentiating cells** | 165 | bootstrapped estimate | 0.689 | 1.061 | 1.018 |
|  |  | p-value | 0.224 | 0.008 | 0.320 |

estimate … bootstrapped ratio of specific linear regression parameter between independent subperiods

p-value … bootstrapped-based significance of test of null hypothesis, that estimate is equal to 1

N … number of independent pairs of observations
